# Knowledge and behaviour of nurse/midwives in the prevention of vertical transmission of HIV in Owerri, Imo State, Nigeria: a cross-sectional study

**DOI:** 10.1186/1472-6955-6-9

**Published:** 2007-10-09

**Authors:** Chizoma M Ndikom, Adenike Onibokun

**Affiliations:** 1 School of Occupational Health Nursing, University College Hospital, Ibadan, Oyo State, Nigeria; 2 Department of Nursing, University of Ibadan, Oyo State, Nigeria

## Abstract

**Background:**

Mother-to-Child Transmission (MTCT) of HIV remains the main mode of acquisition of HIV in children. Transmission of HIV may occur during pregnancy, delivery or breastfeeding. Studies have shown that some specific interventions help to reduce the transmission of the virus to the baby. In order to target safe, rational and effective intervention to reduce MTCT of HIV, it is necessary to ensure that the nurse/midwife has knowledge of the strategies for the prevention of vertical transmission of HIV.

**Method:**

The cross-sectional design was utilized to determine the knowledge and behaviour of nurse/midwives in the prevention of vertical transmission of HIV in Owerri, Imo State, Nigeria. The study sample consisted of 155 nurse/midwives drawn from three selected hospitals through stratified random sampling method. Official permission was obtained from the institutions and consent from participants. Data was collected through the use of a self administered questionnaire. Information sought included respondents' demographic characteristics, knowledge about and behaviour of prevention of vertical transmission as well as factors influencing behaviour.

**Results:**

Findings revealed that nurse/midwives had moderate level of knowledge with mean score of 51.4%. The mean score on behaviour was 52.5%, major factors that influence behaviour in these settings were mainly fear of getting infected, irregular supply of resources like gloves, goggles, sharp boxes, and water supply was not regular also. Hypotheses tested revealed that there is a positive relationship between knowledge and behaviour (r = 0.583, p = 0.00). Knowledge level of nurse/midwives who had educational exposure was not different from those who did not (t = 1.439, p = 0.152). There was a significant difference in the knowledge of nurse/midwives who had experience in managing pregnant women living with HIV/AIDS and those who did not (t = 2.142, p = 0.03). Also, there was a significant relationship between behaviour and availability of resources (r = 0.318, p = 0.000).

**Conclusion:**

The study revealed that the nurse/midwives though moderately knowledgeable still had gaps in certain areas. Their behaviours were fairly appropriate. There is need for improved knowledge through structured educational intervention. Resources needed for practice should always be made available and the environment should be much more conducive for practice.

## Background

Vertical transmission remains the main mode of acquisition of HIV infection in children. A total of 700,000 children were newly infected in 2003, mainly through mother-to-child transmission of HIV [1]. Transmission is rare during early pregnancy and relatively frequent in late pregnancy [2]. According to Musoke and Mmiro [3], "the number of children living with HIV infection is estimated at 13 million with 33.8 million deaths since the epidemic began, each year, approximately 2.4 million infected women give birth and 1800 infants acquire HIV infection every day". WHO [4], stated that "without preventive treatment, up to 40% of children born to HIV-positive women will be infected, majority through MTCT". It is believed that the two-thirds are infected during and around time of delivery and one-third are infected through breastfeeding." Newell [2], opined, "risk factors of vertical transmission include maternal progression of infection, measured as the peripheral blood viral load, or by clinical immunological markers". Premature infants are more likely to be infected than full term infants and the risk increases with the duration of membrane rupture.

Thus, there is need for more effort to be made to prevent transmission of HIV in order to ensure their survival. New survey underscores the disproportionate impact of the AIDS epidemic on women especially in Africa [5]. In 2005, estimated 38.6 million people worldwide were living with HIV [6]. 24.5 million were in Sub-Saharan Africa[5]. Nigeria had 2,900,000 people living with HIV, out of which 1,600,000 were women aged more than 15 years, 240,000 were children aged less than 15 years, while 1,000,000 were men [7]. The rate of new infections will also affect the rate of heterosexual transmission and transmission from mother-to-child.

As the number of children infected during the HIV epidemic increase, the nurse/midwife has an important role to play in ensuring that mother-to-child transmission is reduced to the least possible level. The prevention of mother-to-child transmission of HIV could be reduced most effectively if all available intervention approaches were considered. The major approaches for prevention include, primary prevention of infection among parents to be, preventing unwanted pregnancy among women living with HIV and preventing transmission from HIV-positive women to their offspring, then providing care for HIV-infected mothers and their infants [4].

The nurse/midwives' knowledge of HIV is important because it is the basis on which positive changes in behaviour occur because it brings awareness, which in turn leads to action. Knowledge, training and experience in every aspect of one's profession are very important. Knowledge is operationally defined in the study as the level or degree of information acquired by the nurse/midwife in relation to vertical transmission of HIV/AIDS. On the other hand Practice refers to direct goal oriented actions taken by the nurse/midwife in order to prevent the transmission of HIV from mother to child in the course of her professional duty as a nurse. Behaviour operationally refers to, the actions or reactions of the nurse/midwife, usually in relation to HIV/AIDS prevention and the working environment. Issues relating to HIV/AIDS have generated a lot of fear in the mind of health workers especially nurses and midwives. Some act inappropriately towards HIV seropositive clients. This makes one wonder if they had adequate knowledge about mode of transmission of HIV and adequate knowledge and skill to prevent it. Some studies had already been carried out to determine nurse/midwives knowledge.

Study by Grellier, on midwives' knowledge of HIV virus and its implication for their attitude and practice revealed that, "though training may provide midwives with technical knowledge, it does not necessarily equip them to deal with many of the underlying issues, which in turn reflect on midwifery practice, It is important for practical knowledge to be placed within a broader context" [8]. On the other hand, a study carried out in Lagos State, Nigeria on attitudes of health care providers to persons living with HIV/AIDS by Adebajo, Bamgbala and Oyediran, revealed that "most of the respondents (96.3%) had moderate to good knowledge of HIV/AIDS but the attitude of the nurses towards people living with HIV/AIDS was poor" [9]. Also findings from a study carried out by Mbanga, Sebade, Kegne, Minkuolau and Awah, to assess knowledge and practices of nurses with regards to HIV/AIDS revealed that 70.1% of the nurses who responded scored highly in the knowledge section compared to 50.5% in the attitude and practice section [10].

These studies indicate that knowledge of HIV/AIDS has improved to some extent, though their knowledge did not correlate with their attitude and behaviours. The nurse/midwife has a vital role to play in the achievement of the millennium goals of reduction of child mortality, improving maternal health and combating HIV/AIDS [11]. The nurse/midwives are open systems that receive input, process them to give output, and the feedback returns to her as input [12]. Systems theory is one method of conceptualising the relationship of individuals, their environment and health (Sherwen, Scoloveno and Weignerten, 13). The theory provides a way to understand the many influences on the nurse/midwife, and the possible impact of change of any part on the whole. On the other hand Role theory explains behaviour in terms of the roles, role expectation and demand, role set and reference group operating on the participants in the set of behaviours or function associated with particular position within a particular social context (Wightman and Deux, 14). The nurse/midwife is expected to put her knowledge into practice when she perceives the importance of her role in reducing MTCT of HIV.

Many tertiary institutions like Federal Medical Centre, Owerri have developed formal pattern of incorporating Voluntary Confidential Counselling and Test (VCCT) into the antenatal services. Effort has been made to train nurse/midwives but not all have had this training. Many secondary and primary health institutions in Nigeria are yet to commence this training programme. In some of these institutions, women do not always receive voluntary confidential counselling before HIV test. Many are stigmatised after the test, if found positive. There is need to explore reasons for this. Nurse/Midwives have a lot of role to play in prevention of vertical transmission of HIV, thus, there is need to assess their current state of knowledge and behaviour. The main aim of the study was to determine the knowledge and behaviour of nurse/midwives in the prevention of vertical transmission of HIV in selected hospitals in Owerri, Imo State.

## Methods

The study utilised a cross-sectional design to elicit information on knowledge and behaviour of nurse/midwives in the prevention of vertical transmission HIV. 155 nurse/midwives were drawn from three selected hospitals namely Federal Medical Centre (FMC), General Hospital (GH), Holy Rosary Hospital (HRH), all in Owerri, who have worked for a year in maternity and paediatric units of the hospitals using stratified random sampling method. All cadres were represented. About 50% of respondents from each setting were selected for representativeness.

**The settings **were in Owerri, the capital of Imo state. It is in the South East geopolitical zone of Nigeria. The maternity and paediatric units consist of the following: Antenatal Clinic, Prenatal Ward, Gynaecology Ward, Labour Room, Postnatal Ward, Paediatrics Wards. The Federal Medical Centre was founded in 1995. The total number of nurse/midwives in the Obstetrics and gynaecology and paediatric units in FMC was 130. General hospital Owerri was founded in 1902. There were 111 working in the maternity and paediatric units. Holy Rosary Hospital Emekuku was established in 1933 by Catholic missionaries. There were 69 nurses in the maternity and paediatric unit. Purposive sampling of three hospitals in Owerri was done. These hospitals have all the cadres of nurses working in them and are utilised by the populace. They were the three largest hospitals in the town that serve as referral hospitals for others. Thus, they were found suitable for the study.

**The target population **comprised of all registered nurse/midwives in Imo state. **The study population **includes all nurse/midwives in the selected units in Federal Medical Centre Owerri, General Hospital Owerri and Holy Rosary Hospital, Emekuku-Owerri. The total number of these nurse/midwives in the units was 310.

**The sampling technique **employed was the stratified random sampling technique. The selected hospitals formed the first stratum. The cadres formed the second stratum to ensure that every group is represented. A sample of 155 Nurse/Midwives was selected using stratified random sampling. Since the sampling population was not very large, 50% of respondents from each setting and each cadre were selected. They were selected from all the wards in the obstetric and gynaecology and paediatric units of the three selected hospitals. This gave rise to 65 from FMC Owerri, 55 from GH Owerri and 35 from Holy Rosary hospital Emekuku. The total sample was 155 Nurse/Midwives. The sample size was inflated by 10% to accommodate refusal and incompletely filled questionnaires and those that may not meet the criterion.

### Tabular presentation of participants

Total no of personnel: 310

The randomized no: 180

The total no of respondents: 155

The no of personnel who refused: 25

The final number as a percentage of total personnel: 50%

**The instrument **for data collection was a self administered questionnaire developed by the researcher from reviewed literature. Information sought included respondents' demographic characteristics, knowledge about and behaviour of vertical transmission of HIV prevention, as well as factors that influence behaviour. The tool was divided into four sections.

Section A contained the demographic variables of the subjects and consists of ten items.

Section B consisted of items on knowledge of vertical transmission of HIV. There were twenty items. Thirteen items utilised Yes or No response, six utilised multiple choice options and one was open ended question which dealt with full meaning of HIV.

Section C composed of items on behaviour of prevention of mother-to-child transmission of HIV. There were ten major items used to elicit level of behaviour of prevention of HIV vertical transmission by nurse/midwives in Owerri. Likert's three point scale option, that is always, sometimes, never was used in 5 questions; Yes or No response was used for 5 items.

Section D contained items on factors that influence behaviour of mother to child transmission of HIV prevention. The section contained ten questions and had Yes or No or don't know responses.

The maximum point for a positive response was 3 while the negative response score was 1. There were a total of 50 items.

### Validity of instrument

The instrument was presented to other experts in this field of study and was modified where necessary. The instrument was pre-tested before final data collection.

### Reliability

Test re-test of the research instrument was done before the actual administration of the questionnaire for reliability. The test-retest at a three week interval period yielded a Pearson correlation coefficient of 0.81. The test confirmed that the instrument was suitable for the study.

### Ethical consideration

An introductory letter from the Department of Nursing, University of Ibadan and an application letter for permission, stating the nature of study was presented to the hospital authorities in the selected setting in order to obtain permission.

The letters were then presented to their ethical committees who reviewed the research instrument before a written permission was given.

Consent of participants was sought and their right to participate or not to participate was respected. They were informed that they had the right to withdraw from the exercise if they wish to. The questionnaire also had an informed consent form on the front page which they had to sign first before filling the questionnaire.

### Procedure for data collection

The data was collected by the researcher with the assistance of ward managers and research assistants. The questionnaire was given to the respondents to fill after obtaining informed consent. The respondents were assured of anonymity and confidentiality of information. The first stage of data collection was to compile the list of all the nurses on the wards in the maternity and children's ward. Stratified random sampling method was used to select respondents.

The selected respondents were given the questionnaire, trained research assistants helped in the distribution of questionnaires. A period of one month was used for distribution and retrieval of the questionnaires.

### Procedure for data analysis

Filled questionnaires were analysed using Statistical Package for Social Sciences (SPSS) software. Descriptive statistics were presented as graphs and tables to show frequency distribution of scores as well as their percentages. Cross tabulations and chi square statistics were used to determine the relationship between behaviour and demographic variables since they formed categories. The items on knowledge and behaviour were coded and scores were given. Coded scores of 1 to 3 were used and were later converted to percentages. The scale of measurement was interval as there was no 0 point. Thus Pearson Correlation and students't-test were found suitable for the statistical analysis. Four null hypotheses were stated at the commencement of the study and they were tested using Pearson Correlation to measure relationships while student t test was used to compare the differences between two means.

## Results

### Demographic characteristics of respondents

The study was carried out in Owerri with 155 respondents, 65 (42%) were from FMC, 55 (35%) were from GH and 35 (23%) were from HRH. Majority (24%) of the respondents had a mean age of 37.3 years and were married (76.8%), with diploma as their highest educational qualification (81%). Also, they were mainly registered nurse/midwives only (84%) and had 14.5 years as mean post qualification experience (27.7%) and 5 years as mean of their experience in obstetrics and gynaecology unit; their first source of information on HIV/AIDS was workshops/seminars (60.3%), 41% received lectures on HIV as students while 42.2% have ever managed pregnant women living with HIV as presented on table 1.

**Table 1:** Demographic characteristic of respondents

**Variable**		**frequency (%)**	** mean (SD)**
Age(years)			37.3(8.7)
Marital status	married	119(76.8)	
Professional qualification	RNM only RNMPH RNM others	130(84) 16(10) 9(6)	
Educational qualification	Diploma	125(81)	
Designation	Nursing Officer	46(29.7)	
Post qualification years of experience (years)			14.5(9.3)
Year of experience in maternity unit(years)			05(6.6)
First source of information on HIV	Workshop/Seminar	85(60.3)	
Received lecture as a student on HIV		64(41.0)	
Managed a pregnant woman living with HIV		67(42.2)	

### Respondents' knowledge of vertical transmission of HIV

Majority (91%) of respondents were aware of vertical transmission of HIV. Also, majority (67%) had knowledge of the full meaning of HIV. Majority (89%) identified the class of the virus as retrovirus, and had affinity for CD4 receptors (78.7%). On the other hand, (36.8%) of the respondents could not correctly identify reverse transcriptase as the enzyme used to copy RNA to DNA. Also, (45.8%) of the respondents did not know the normal CD4 count. Only 19% of the respondents identified the confirmatory test as western blot as seen on table 2.

**Table 2:** Individual items and respective scores assessing knowledge of HIV with percentage of correct responses

**Items**	**Correct answer**	**Score**	**Frequency (%)**
Name of the virus	Human Immunodeficiency Virus	3	**44(28.4)**
Class of virus	Retrovirus	3	**138(89)**
Virus affinity	CD4 receptors	3	**122(78.7)**
Enzyme used to copy RNA to DNA	Reverse transcriptase	3	**41(26.5)**
Normal CD4 count	500 – 1200 cells/mm	3	**64(41.3)**
Drug commonly use in PMTCT	Nevirapine	3	**98(63.2)**
**Risk factors in MTCT:**			
Artificial rupture of membranes	yes	3	**57(36.8)**
Caesarean section	no	3	**40(25.8)**
Perineal trauma	yes	3	**89(57.4)**
Vaginal delivery	yes	3	**92(59.4)**
**Strategies for PMTCT:**			
Use of antiretroviral drugs	yes	3	**93(60)**
Exclusive formula feeding	yes	3	**91(58.7)**
Voluntary counselling and testing	yes	3	**91(58.7)**
Caesarean section option	yes	3	81(52.3)

The respondents demonstrated knowledge of strategies for prevention of HIV as they agreed on the use of antiretroviral drugs (60%), exclusive formula feeding (58.7%), voluntary counselling and testing (58.7%), caesarean section option (52.3%). Also, 88.4% of respondents agreed that universal precautions should be used with all the patients, at all times regardless of diagnosis. Mean score on knowledge was 51.4%. Majority of the respondents (51%) had moderate level of knowledge that is between 48 and 59 while 34.2% had low level of knowledge that is 47 and below as measured by this knowledge test.

### Respondents' behaviour of MTCT prevention strategies

Majority of the respondents, 78.1% always educate women on HIV/AIDS, only (37.4%) offer VCT to clients, while 76.8% always counsel women on safe infant feeding practices and majority (81.3%) use aprons, gloves and masks during delivery as seen on Table 3.

**Table 3:** Respondents' behaviour of MTCT prevention strategies

**Item**	**Positive response**	**Score**	Frequency (%)
How often do you educate women on HIV/AIDS?	always	3	**121 (78.1)**
How often do you offer voluntary counselling and testing (VCT) to pregnant women?	always	3	**58 (37.4)**
How often do you obtain consent before testing?	always	3	**87(56.1)**
Are the regnant women usually Counselled with their husbands?	always	3	**55(35.5)**
How often are women counselled on safe infant feeding?	always	3	**119(76.8)**
Do you use aprons, gloves and masks during delivery?	yes	3	**126(81.3)**
Do you encourage mothers living HIV to feed baby exclusively with formula?	yes	3	**139(89.7)**
Do you gloves when checking soiled sanitary pad?	yes	3	**147(94.4)**
**In case of accidental exposure to contaminant during Delivery :**			
Do you wash under running water?	yes	3	**126(81.3)**
Do you report the incident?	yes	3	131(84.5)

Generally, behaviour was seen as fairly appropriate. Cross tabulations showed that age and post qualification years of experience of respondents had significant relationship with behaviour on tables 4 and 5. Majority (47%) had moderate score on behaviour while 35.5% had low score on behaviour. Mean score on behaviour was 52.5%.

**Table 4:** Relationship between demographic variables and behaviour Chi-square test of relationship between respondents' age and behaviour only

	Age									
**Behaviour**	20–25	26–30	31–35	36–40	41–45	46–50	50+	Total	χ^2^	**P < 0.05**

≤ 47	4	10	3	8	15	9	6	55	244.65	**0.002**
48–59	5	18	6	15	18	8	3	73		
≥ 60	3	10	3	5	3	1	2	27		

**Total**	**12**	**38**	**12**	**28**	**36**	**18**	**11**	**155**		

**Table 5:** Chi-square test of relationship between respondents' years of post qualification experience and behaviour

	Years of post qualification experience									
**Behaviour**	1–5	6–10	11–15	16–20	21–25	26–30	30+	Total	χ^2^	**P < 0.05**

≤ 47%	15	6	3	6	11	13	1	55	192.36	**0.005**
48 – 59%	19	6	14	18	9	6	1	73		
≥ 60	9	7	2	1	3	5	-	27		

**Total**	**43**	**19**	**25**	**23**	**24**	**24**	**2**	**155**		

### Factors that influence behaviour of MTCT of HIV prevention

The study revealed that 36.1% agreed that time constraint did influence their behaviour. Other factors include willingness of women to be tested (80.6%), confidence in educating clients (90.3%), fear of contagion experience while caring of pregnant women living with HIV (76.8%), resources needed like gloves were not always available (62.6%), water supply not regular (43.9%), support from clients' husband (45.8%) and support from professional colleagues (80%) – Table 5. Other resources like goggles were not available in some hospitals; sharp boxes were not available in the institutions at the time of the study.

### Hypotheses testing

Tables 6, 7, 8, to 9 showed the results of tested the Hypotheses. The first Hypothesis showed a significant relationship between knowledge and behaviour of prevention of vertical transmission of HIV among the respondents (r = 0.538, p = 0.000). The second showed that there is no significant difference between the knowledge of respondents that had educational exposure on HIV and those who did not (t = 1.439, p = 0.152). Hypothesis three showed a significant difference between the level of knowledge of those who had experience in the care of pregnant women living with HIV and those who did not (t = -2.142, p = 0.03). Availability of resources for HIV prevention had a significant relationship with behaviour as seen in the result of the fourth hypothesis (r = 0.318, p = 0.003), Table 10.

**Table 6:** Respondents' opinion on factors that influence behaviour

**Factors**	Yes
Are the women willing to be tested in your hospital?	**125 (80.6%)**
Are you sometimes unable to give care desired because of time constraint?	**56 (36.1%)**
Do you feel confident educating clients on issues relating to HIV/AIDS?	**140 (90.3%)**
Do you experience fear of contagion when caring for pregnant women living with HIV?	**119 (76.8%)**
Do you receive regular supply of gloves in your unit?	**97 (62.6%)**
Do you have regular supply of water in your unit?	**68 (43.9%)**
Are the clients' husbands willing to give support to their wives during treatment?	**71 (45.8%)**
**Do other professional colleagues give their support?**	**124 (80.0%)**

**Table 7:** Hypothesis 1. Relationship between knowledge and behaviour

**Variables**	**N**	**Mean**	**r**	**P < 0.05**	**Remark**
Knowledge	155	51.4		0.00	Significant
Behaviour	155	52.5	0.538		

**Table 8:** Hypothesis 2. Knowledge and exposure to information on HIV

					**95% confidence interval of the difference**					
									
	**Seminar/workshop**	**N**	**Mean**	**SD**	**Lower**	**Upper**	**t**	**df**	**P < 0.05**	**Remark**
Knowledge	Yes No	85 70	51.7 50.4	6.28 5.32	-3.23	0.51	-1.439	150	0.152	Not significant

**Table 9:** Hypothesis 3. Knowledge and experience of managing pregnant women living with HIV

					**95% confidence interval of the difference**					
									
	**Ever Managed a pregnant woman**	**N**	**Mean**	**SD**	**Lower**	**Upper**	**t**	**df**	**P < 0.05**	**Remark**
Knowledge	Yes No	64 88	52.6 49.3	5.60 5.84	-3.23	0.51	-2.142	150	0.03	significant

**Table 10:** Hypothesis 4. Behaviour and availability of resources

**Variables**	**N**	**r**	**P < 0.05**	**Remark**
Behaviour Resources	155 155	0.318	0.00	Significant

## Discussion

The general objective of this study was to examine the knowledge and behaviour of prevention of vertical transmission among nurse/midwives and to determine if their behaviour is related to knowledge or influenced by other factors. It is interesting to note that they are aware of vertical transmission of HIV and majority (60.3%) became aware through workshop/seminar attendance for the first time and only 41% received lectures as students. HIV/AIDS as a disease was documented in the 1980s and was added to nursing curriculum in the 1990s, thus information on HIV was passed on mainly through seminars and workshops. This was supported by UNAIDS, report on a study carried out in University of Witwatersrand in South Africa, which revealed that many nurses qualified before HIV/AIDS was in the curriculum [16]. Despite this, the study revealed that a significant proportion of the respondents had moderate level of knowledge as measured by the knowledge test with mean score of 51.4%. However, there existed many gaps in the knowledge of HIV as over 50% of respondents could not correctly answer the items on pathophysiology, diagnostic tests, and factors that influence vertical transmission. In line with this finding, Adebajo et al [9], noted from a study of nurses in Lagos State, that "significant proportion, 96.5% of the respondents had appreciable (moderate to high) scores, however, in spite of this, there existed many gaps in their knowledge of HIV, they had many misconceptions regarding how HIV/AIDS can be transmitted". The result is consistent with the study carried out by Mbanga et al [10], to assess knowledge and practice of nurses in regard to HIV/AIDS in Cameroon which revealed that 70% of the nurses scored highly on the knowledge of HIV.

The hypothesis on knowledge and educational exposure (t = -1.439, p = 0.152) showed that there is no significant difference between those that had educational exposure through workshop and seminar and those who did not. The findings were supported by Bennet and Weale [17] which revealed that "awareness training programme did not make any significant difference in the knowledge and attitude between those that attended the programme and those who did not". They suggested the need to review HIV related training for midwives. The hypothesis on difference between knowledge of those who had experience in the management of pregnant women living with HIV and those who did not (t = -2.142, p = 0.03) on table 9 showed a significant difference in their level of knowledge. This shows that majority acquired their knowledge when they managed such clients. The experience of managing the clients enabled them to understand the condition better. Clinical experience is still very important in nursing as experience had lasting impact on the knowledge of the nurse/midwives.

The relationship between knowledge and behaviour was tested as hypothesis 1 using Pearson correlation, on table 7, revealed a significant positive relationship (r = 0.538, p = 0.000). This indicates that there is a strong positive relationship between knowledge and behaviour. Training improved midwives knowledge and this in turn improved their understanding of HIV testing policy and offering of testing to all pregnant women [18]. Though many factors could make a difference between what is known and what is done [10], yet the respondents with higher level of knowledge also scored better in area of behaviour since one cannot carry out what she does not know.

A significant positive relationship existed between availability of resources and behaviour of nurse/midwives in vertical transmission prevention (r = 0.318, p = 0.00), Table 10. The respondents were sometimes unable to practice what they knew because what they require for practice was not available. This was supported by chambers et al [19], as they discovered that, "multiple reasons were offered for current practice, including perceived reluctance by women to be tested, lack of time, skills, knowledge and support services". The study revealed that majority – 119 (76.8%) experience fear when rendering care, 81 (52.3%) said supply of water was not regular. These two factors could also negatively affect behaviour. Fear of contagion was a major factor affecting behaviour. The nurses feel their life is at risk and the resources needed to protect themselves were not always available. Fear is the most obvious factor influencing behaviour negatively. It contributes to stigmatization and negative attitude. The fear of being infected at workplace, educational institutions and in community has led to irrational discriminatory treatment of people living with HIV/AIDS [9].

The researcher observed that the nurses found it difficult to practice in some cases due to insufficient supply of gloves, which makes the use of a pair of gloves longer than necessary. Also, face masks, goggles, sharp boxes were not always available. Every institution is required to have goggles and sharp boxes, but these were conspicuously absent in some of the institutions, even gloves were not supplied in large quantities, which made the practitioners to re-use gloves. Water supply was not regular. The nurse/midwife continues to practice in fear since she cannot protect herself adequately. Needles were being recapped since there were no sharp boxes. Thus the practice of prevention of vertical transmission is being influenced by limited supply of resources. Standard for infection control has to be met for behaviour to be appropriate.

## Conclusion

The study revealed that the Participants answered approximately 50% of the items correctly on the HIV knowledge test. Knowledge was acquired as an input from the environment, through lectures, workshops, seminars and experience. Major factors that influenced behaviour in these settings were mainly fear of contagion, irregular supply of resources like gloves, goggles, sharp boxes and water. The practice of VCT was not very good as only 56.1% of respondents reported that they always obtained consent before VCT. This is not a very good practice because testing women without their permission is unethical. Also fear of contagion results in stigmatization.

Correct input in the right environment will ensure correct output and effective behaviour. Thus, it can be deduced that the nurse/midwives were partially effective in the practice of prevention of vertical transmission of HIV, since they had moderate level of knowledge and resources for role performance were not always available. There is need for improved knowledge through structured educational intervention. Resources needed for practice should always be made available and the environment should be made more conducive for practice. More elaborate studies should be carried out in this field in other parts of Nigeria in order to ensure improvement in knowledge and behaviour.

## Abbreviations

AIDS: Acquired Immune Deficiency Syndrome.

DNA: Deoxyribonucleic acid

FMC: Federal Medical Centre

FMOH: Federal Ministry of Health

GH: General Hospital

HIV: Human Immunodeficiency Virus

HRH: Holy Rosary Hospital

MTCT: Mother-to-Child transmission

RNA: Ribonucleic acid

UN: United Nation

VCCT: Voluntary Confidential Counselling and Testing

VCT: Voluntary Counselling and Testing

WHO: World Health Organisation

## Competing interests

The author(s) declared that they have no competing interests.

## Authors' contributions

CN did the study under the supervision of AO. Both authors have read and approved the final version of the manuscript.

## Pre-publication history

The pre-publication history for this paper can be accessed here:


